# Using a Learning Health System to Integrate Peer Support in Early Intervention Services for Psychosis in Quebec: Protocol for a Participatory, Mixed‐Methods Study (the PAIRPEP Project)

**DOI:** 10.1111/eip.70092

**Published:** 2025-10-14

**Authors:** Beatrice Todesco, Srividya N. Iyer, Manuela Ferrari, Marc‐André Roy, Marie‐Hélène Morin, Julie Marguerite Deschênes, Camille Arbaud, Gabriel Julien, Annie Bossé, Mary Anne Levasseur, Amal Abdel‐Baki

**Affiliations:** ^1^ Neurosciences Axis, Research Center of the Centre Hospitalier de l'Université de Montréal (CRCHUM) Quebec Canada; ^2^ Department of Psychiatry McGill University Quebec Canada; ^3^ Department of Psychiatry and Neurosciences, Faculty of Medicine Université Laval Quebec Canada; ^4^ Department of Psychosociology and Social Work Université du Québec à Rimouski (UQAR) Rimouski Québec Canada; ^5^ Association québécoise pour la réadaptation psychosociale Quebec City Québec Canada; ^6^ Department of Psychiatry and Addiction, Faculty of Medicine Université de Montréal Quebec Canada

**Keywords:** early intervention services for psychosis, implementation, learning health system, mixed methods, peer support, youth mental health services

## Abstract

**Introduction:**

Since 2019, SARPEP (Système Apprenant Rapide pour les programmes de Premiers Épisodes Psychotiques), a rapid learning health system (RLHS) for Quebec's Early Intervention for Psychosis Services, operates to bridge the evidence‐practice gap across the province. Despite strong stakeholder support and government recommendations, peer support services remained poorly available. To address this gap, since 2023, the PAIRPEP project was co‐developed to support and evaluate the implementation of peer support and family peer support. This paper describes the co‐designed study protocol, embedded within this RLHS.

**Methods:**

This participatory, mixed‐methods study aims to examine the implementation of the PAIRPEP intervention longitudinally over 3 years across 12 Early Intervention Services and its impact on multiple stakeholders. Informed by the Medical Research Council framework for complex interventions, the project includes a co‐designed (with multiple stakeholders) multimodal capacity‐building program with specific components developed to overcome barriers to integrating peer support and family peer support. Quantitative questionnaires are collected every 4 months from clinicians while youth and families can complete surveys at any convenient time, via QR codes available in clinics, through the RLHS electronic platform. Focus groups are conducted annually over 3 years with eight stakeholder groups. The analysis integrates findings using thematic synthesis and joint displays to assess convergence and divergence across methods and perspectives.

**Results and Conclusion:**

This protocol paper outlines the study's co‐design, procedures and anticipated contributions. Embedding large‐scale innovative intervention implementation (such as peer support) within an RLHS can foster real‐time feedback, iterative refinement and inform clinical practice and policies.

## 1 Introduction

### 1.1 Peer Support in Mental Health Services: Context and Rationale

Peer support is grounded in the belief that individuals who have confronted and overcome adversities can offer encouragement, hope and mentorship to others facing similar challenges (Davidson et al. 2006). Initially informal and mutual, peer support has gained formal recognition in recent decades as part of a recovery‐oriented approach in mental health services (Davidson et al. 2012; Smit et al. 2023). A growing body of evidence supports the benefits of peer support interventions for personal recovery (Lloyd‐Evans et al. 2014; Lyons et al. 2021; White et al. 2020), with a recent meta‐analysis showing a small yet significant effect size on clinical and functional recovery (Smit et al. 2023).

International agencies/organisations have advocated for broader dissemination of peer interventions, developing policies and frameworks to support the integration of peer support workers (PSWs) into mental health services (Council of Australian Government Health Council 2017; Health Education England 2017; WHO 2021). International initiatives such as Orygen (Australia), NAVIGATE (RAISE‐ETP/EPI‐NET network, USA), OnTrackNY (New York, USA) and Mindgardens (Australia) have integrated peer support in early intervention services (EIS) for psychosis through structured training and supervision frameworks.

In Canada, initiatives such as Foundry (BC) and Support House (Ontario) have laid the groundwork for a more structured and contextually responsive integration of peer support within Canada's youth mental health services, including Quebec's Early Intervention for Psychosis Services (EIS).

In Quebec, mental health action plans of the health and social services ministry (MSSS) (Delorme and Breton 2005; Delorme et al. 2015; MSSS 2022a) and the *Cadre de reference pour les programmes pour premiers épisodes psychotiques* (provincial guidance for EIS) (Delorme et al. 2017; MSSS 2022b) recommended the inclusion of PSWs within clinical teams. Despite governmental orientations, their integration remains limited in EIS (Abdel‐Baki, Ferrari, et al. 2025; Bertulies‐Esposito et al. 2020; Ferrari et al. 2025).

Successful integration requires strong advocacy, well‐defined policies, adequate funding, proper training and certification, team preparedness and communication, clear role description and the promotion of a recovery‐oriented service culture (Ibrahim et al. 2020; Mirbahaeddin and Chreim 2022; Pires de Oliveira Padilha et al. 2023). The well‐being and self‐care of peer workers are essential for seamlessly integrating the peer workforce into these teams (Ibrahim et al. 2020; Mirbahaeddin and Chreim 2022).

Research on PSWs in EIS remains limited, predominantly relying on exploratory qualitative studies (Nguyen et al. 2022). Interviews with UK‐based PSWs in EIS highlighted PSWs' roles in destigmatizing psychosis, reducing social isolation, and serving as symbols of hope (Nguyen et al. 2022). However, integrating PSWs into EIS teams is complex, often requiring considerable time for mutual adjustment (Simmons et al. 2020; Hopkins et al. 2021). PSWs face significant job‐related uncertainties, undergoing a months‐long adaptation phase to build confidence and trust in their roles (Simmons et al. 2020). Even if the clinical team is well disposed towards PSWs, uncertainties can arise regarding role ambiguities and the team dynamics related to the unique position of PSWs (Hopkins et al. 2021).

Overall, key questions persist regarding the necessity for specialized training, the most effective delivery settings, the optimal structuring of peer interventions and the overall impact of these interventions within the EIS framework (Nguyen et al. 2022; Pires de Oliveira Padilha et al. 2023).

### 1.2 The SARPEP Learning Health System: A Unique Implementation Context for Peer Services

Since 2019, the rapid learning healthcare system “Système Apprenant Rapide pour Premiers Épisodes Psychotiques” (SARPEP) has been introduced into Quebec's EIS network to enhance the quality of care by bridging the evidence‐practice gap (Ferrari et al. 2022). SARPEP includes a digital platform that enables real‐time data collection, feedback sharing and monitoring of service delivery, patient outcomes, users' satisfaction and sharing among EIS (Ferrari et al. 2022). Continuous data collection offers ongoing insights into the progress of implementing EIS components over time and allows for direct comparisons among similar programs. Regular feedback on data is electronically generated, providing tailored suggestions for improvement to each EIS. Furthermore, a community of practice, comprising multiple stakeholders including youth, family members, clinicians, team leaders, managers, researchers and policymakers, provides access to training and clinical or administrative tools based on the identified needs.

From late 2019 to January 2024, SARPEP brought together 20 EIS within 14 integrated health and social services centres, involving more than 170 healthcare professionals, 60 psychiatrists and 20 team leaders, who altogether offer services to 2700 patients per year along with their families. With its participatory nature, focus on quality care and ability to generate accessible feedback and support while fostering a community that brings together diverse stakeholders—all actively involved in intervention development and delivery—the SARPEP community represents a uniquely suitable ecosystem for implementing and refining EIS components.

Feedback from SARPEP highlighted the poor availability of peer services within EIS (Abdel‐Baki, Ferrari, et al. 2025). To address this gap, a targeted 3‐year initiative called “PAIRPEP” (“PAIR‐Aidance pour les Premiers Episodes Psychotiques”) was launched to build capacity and promote the integration of PSWs and Family peer support workers (FPSW) in 12 selected EIS across diverse settings of the Quebec province (Abdel‐Baki et al. 2024).

### 1.3 Conceptual Framework: Developing and Evaluating Complex Interventions

The project is grounded in the updated Medical Research Council framework for developing and evaluating complex interventions (Skivington et al. 2021), which outlines four interrelated and overlapping phases: (1) intervention development, (2) feasibility assessment, (3) intervention evaluation and (4) implementation (Skivington et al. 2021). The Medical Research Council framework emphasizes six core elements requiring continuous reassessment throughout the implementation process, which are detailed in Table 1 as adapted and applied to the PAIRPEP project.

**TABLE 1:** Core elements of the Medical Research Council framework for complex interventions applied to the PAIRPEP initiative.

Core elements of the Medical Research Council framework for complex interventions applied to the PAIRPEP project
*CONTEXT: Dynamic and reciprocal interactions between the intervention and its context, with each influencing and transforming the other* Analyse variations in the context in which PAIRPEP operates across different sites (e.g., clinic location—rural or urban, available resources, prior engagements with PSWs/FPSWs, team readiness and attitude towards the “lived experience” paradigm, socio‐political context, etc.). Longitudinally investigate how contextual factors impact the acceptability and effectiveness of the PAIRPEP multi‐modal capacity building program over time.
*PROGRAMME THEORY: Ongoing refinement of program theory, informed by real‐world observations and iterative adjustments to the intervention or its context* Identify the necessary steps, elements and processes involved in supporting implementation of peer support within EIS, from the recruitment and training of PSWs/FPSWs to their integration within clinical teams and refine the PAIRPEP program over time.
*STAKEHOLDERS: Continuous engagement with stakeholders throughout the development and evaluation of the intervention to optimise positive outcomes* Throughout all project phases, continuously evaluate stakeholder involvement (including PSWs, FPSWs, clinicians, managers, service users and family members) at all levels, ensuring genuine and meaningful engagement from daily clinical practice to PAIRPEP project development and decision‐making.
*KEY UNCERTAINTIES: Identification of uncertainties through the research process, including emerging questions and areas of ambiguity* Continuously assess what we've learned about supporting implementation of peer support: what do we know and what is still unclear? PAIRPEP capacity building program was created to support implementation of peer support by addressing barriers and enhancing facilitators, based on existing literature. However, we're still figuring out how peer support in EIS compares to what is known in broader peer support literature.
*INTERVENTION REFINEMENT: Iterative refinement of the intervention, with each development phase necessitating adjustments to enhance its acceptability, feasibility and effectiveness* Identify critical areas and issues in our PAIRPEP capacity building program needing redefinition and refinement. Examine how the integration of PSWs with the PAIRPEP model impacts the broader early psychosis and peer support ecosystems of the province over time, involving diverse communities of professionals and patients.
*ECONOMIC CONSIDERATIONS: Ongoing assessment of resources required for the implementation and sustainability of the intervention, alongside evaluating its outcomes and impact*. Examine the challenges and resources, ranging from individual program levels to provincial levels, required to sustain and scale up the implementation of the PAIRPEP program and the integration of PSWs and FPSWs across all EIS in Quebec.

### 1.4 PAIRPEP: A Capacity‐Building Initiative for Peer Support Integration in EIS

PAIRPEP was developed to facilitate the integration of PSWs/FPSWs within EIS by addressing structural, organisational and cultural barriers. The project is structured in three phases: (1) co‐design and consensus‐building to develop the intervention (PAIRPEP multimodal capacity building program) components; (2) collaborative development of evaluation strategies and assessment tools to monitor implementation of the PAIRPEP program and (3) implementation of peer support services across 12 EIS, accompanied by a participatory, mixed‐method evaluation of the process.

**FIGURE 1:**
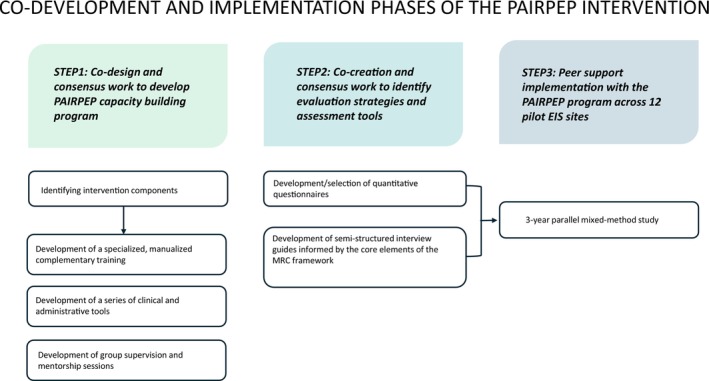
Co‐development and implementation phases of the PAIRPEP intervention. EIS, early intervention services; MRC, Medical Research Council.

The overall research project pathway diagram is summarised in Figure 1. While this article focuses on the research component, another article details the co‐development process of the PAIRPEP capacity building program and its components (Abdel‐Baki, Deschênes et al. 2025). A brief overview of the PAIRPEP program is provided to contextualise the present study.

The PAIRPEP program (also called the PAIRPEP intervention) is a multicomponent program co‐designed to support peer service implementation from recruitment and training to clinical integration and ongoing support. The program is intentionally designed for continuous refinement and enhancement, aiming to adapt to evolving needs by developing new tools and shaping diverse activities or refining existing components. PAIRPEP components are as follows:

1. A manualized complementary training tailored for PSWs/FPSWs working in EIS, providing foundational knowledge specific to this clinical context. All peer workers must have completed certified generic peer support training prior to this EIS‐specific training.
2. Clinical and administrative tools to guide peer support practices and integration of PSWs/FPSWs in EIS. It includes job offer templates; *Integration Checklists* tailored for PSWs/FPSWs, team leaders and managers; a *Summary of Roles and Tasks* for PSWs/FPSWs in EIS and a *Reference and Follow‐Up Form* designed to guide the integration of peer support/family peer support into the service users' recovery plan and note taking for the patient file.
3. Bimonthly group supervision and mentorship sessions led by experienced PSWs/FPSWs to provide continuous peer‐to‐peer guidance, support and learning opportunities. Group peer supervision sessions focus on sharing experiences, discussing complex cases and difficult situations, and addressing challenges encountered in clinical practice. Mentorship sessions, co‐facilitated by expert EIS clinicians and peer mentors, provide targeted support on specific topics related to peer and family peer support work in EIS settings.
4. Community of practice, involving dedicated sessions focusing specifically on challenges related to peer support integration in EIS, as they emerge. The community of practice fosters continuous improvement by ongoing exchange, collaborative problem‐solving and shared learning among all stakeholders.

### 1.5 Study Aims and Research Objectives

The present study aims to evaluate the implementation and impact of PAIRPEP's capacity‐building program on (1) the integration of peer support services and (2) the integration of PSW/FPSW within 12 EIS in Quebec. The specific research objectives are to evaluate, from each stakeholder's perspective:

1. the implementation of PAIRPEP capacity building program, as well as its impacts on the integration of peer support/family peer support intervention in EIS
2. the integration process of PSW/FPSW within EIS teams, as well as their impacts on the stakeholders involved.

These objectives are closely interconnected, as building capacity for peer support integration within the EIS community is expected to drive the successful implementation of this EIS component while also allowing for an in‐depth understanding of its real‐world effects and challenges. Additionally, this study is informed by national and international implementation efforts for integrating peer support, including Foundry in British Columbia (Barbic et al. 2024), Orygen in Australia (Monson et al. 2015), OnTrackNY in the United States (Jones 2015) and Mindgardens in Australia (Singh et al. 2025). While these are not formal guidelines, they offer practical frameworks, tools and lessons learned that have informed the methodological choices and implementation strategies employed in this study, ensuring alignment with evidence‐based and contextually relevant approaches.

## 2 Methods

The study uses a mixed‐method design capturing both quantitative indicators and qualitative experiences from multiple stakeholders, ensuring a comprehensive evaluation of PAIRPEP's implementation process.

### 2.1 Study Design

**FIGURE 2:**
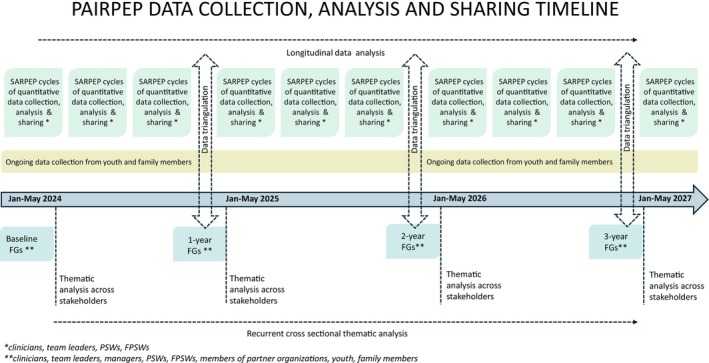
PAIRPEP data collection, analysis and sharing timeline. FG, focus groups; FPSWs, family peer support workers; PSWs, peer support workers.

This research employs a participatory, longitudinal convergent mixed‐method design to examine convergence, divergence and complementarities between quantitative and qualitative data. Guided by the Medical Research Council framework for complex interventions (Skivington et al. 2021), it emphasises contextual adaptation and stakeholder's involvement early on and throughout, particularly clinicians and patients. By integrating multiple stakeholder perspectives, the study aims to provide rich and nuanced insights into the process of implementing peer support within EIS. The overall study process is presented in Figure 2.

### 2.2 Study Settings

The PAIRPEP project involves 12 EIS, which were the 11 sites from the SARPEP pilot project (Ferrari et al. 2022), with one having split due to increased volume and its large catchment area. The sites vary in team composition and size, academic affiliation, and socio‐geographical context (rural/urban, size of catchment area, specific population characteristics such as proportion of immigrants, First Nations youth, youth experiencing homelessness). These EIS's ongoing engagement with all components of the SARPEP LHS, including data collection and participation in the community of practice, makes them ideal settings for implementing and refining peer support.

The 3‐year study is funded by philanthropic partners, from June 2023 to December 2026. This funding covers the salaries of PSWs/FPSWs offering new services within the 12 EIS programs (15 h/week), as well as fees for the accredited basic training of PSWs/FPSWs. In EIS where previous dedicated budgets for hiring PSWs/FPSWs were already in place, the PAIRPEP funding helped add new services to existing resources.

### 2.3 Eligibility and Recruitment Processes for PSW and FPSW

As part of the PAIRPEP project, eligibility for PSW and FPSW roles is based on a combination of experiential and educational requirements. PSWs are required to have lived experience with psychosis and recovery, while FPSWs need experience supporting a loved one affected by psychosis. Candidates are required to complete one of the 2 accredited peer support training programs in the province: the Microprogram in Peer Support offered by *Université de Montréal* or the training offered by the non‐profit organisation, *Association Québécoise de readaptation psychosociale* (*AQRP*) (in collaboration with Université Laval). A post‐secondary diploma in another related field is recommended, although not mandatory. Recruitment follows the established human resources policies of each participating EIS's health institution, which vary a little between centres, although mainly similar. Positions are generally advertised through institutional channels, and in close collaboration with the accredited peer support training programs, to identify and match qualified candidates to the EIS. Some EIS also identify potential candidates themselves, including former patients/family members who, motivated by their lived experience, express interest in pursuing a career in peer support/family peer support. As part of PAIRPEP, all PSWs and FPSWs receive ongoing training and professional development to support their integration into EIS.

### 2.4 Study Participants

Participants are purposefully selected for their direct experience with implementing peer support (Palinkas et al. 2015). Diverse stakeholder groups are recruited to ensure rich multifaceted data. Eligibility and exclusion criteria are detailed in Table 2. The quantitative and qualitative components include participants from the following stakeholder groups: (1) PSWs; (2) FPSWs; (3) Youth receiving EIS; (4) Family Members/Loved Ones of youth receiving EIS; (5) EIS clinicians; (6) EIS team leaders; (7) EIS Managers; (8) Policymakers and (9) Leaders of peer support partner organisations. EIS Managers, policymakers and leaders of peer support partner organisations are only included in the qualitative component. Although these last groups are less directly involved in the day‐to‐day clinical routine of EIS, they play important roles in the integration of PSWs/FPSWs from the hiring process to integration retention. Therefore, their insights into structural, organisational and administrative factors influencing peer support implementation and the transition of PSWs/FPSWs from community‐based organisations to health institutions are essential.

**TABLE 2:** Inclusion and exclusion criteria.

Inclusion criteria
Being part of one of the mentioned key stakeholder groups: PSWs: Certified PSWs employed in one of the 12 EIS participating in the project FPSWs: Certified FPSWs employed in one of the 12 EIS participating in the project Users: Young person experiencing FEP receiving peer support service during the last year in one of the 12 EIS participating in the project Family Members/Loved Ones: Family members or loved ones of youth experiencing a FEP receiving family peer support services during the last year in one of the 12 EIS participating in the project EIS clinicians: Clinical staff members (physicians, case managers, nurses, social workers, etc.) employed in one of the 12 participating EIS who have collaborated with PSWs and/or FPSWs EIS team leaders: Team leaders of one of the 12 EIS participating in the project EIS Managers^a^: Local program managers overseeing operations in one the 12 EIS participating in the project. Members of peer support partner organisation^a^: Leaders of peer support community organisations that are participating in our community of practice and involved in co‐construction of diverse parts of the project. Being capable and willing to provide informed consent for participation in the study Being 18 years of age or older Being proficient in French and able to participate in a group discussion

Exclusion criteria
Participants who do not fall into one of the previously stated 8 categories or who do not meet the other inclusion criteria are not eligible for the study. EIS clinicians/team leaders/managers who have only recently joined the team in the last weeks/months and therefore may not yet be familiar with the service organisation will not be included in the study. Additionally, exclusion criteria for youth hindering acceptance into an EIS program include being diagnosed with moderate to severe intellectual disability.

Abbreviations: EIS, early intervention service; FEP, first episode psychosis; FPSW, family peer support workers; PSW, peer support worker.

^a^ Only applicable to the qualitative research component.

### 2.5 Recruitment and Sampling

#### 2.5.1 Quantitative Component

All team leaders, clinicians and PSWs/FPSWs from the 12 EIS sites are invited by email to complete the quantitative questionnaires as part of the SARPEP RLHS. To encourage broad participation among youth and family members, posters with QR codes linked to the survey are displayed in all EIS, allowing service users (youth and families) to complete the anonymous questionnaires. As part of their routine practice, PSWs/FPSWs and clinicians can invite those receiving peer support or family peer support services by giving them cards with a QR code or a weblink by email to provide their evaluation of their recovery and the impact of services on it and their satisfaction with services received. The questions are about various services, including peer support. In inviting service users and families to fill these questionnaires, the opportunity to help the EIS continuously evaluate itself and improve is emphasized. Participation is voluntary, and each EIS site holds a draw every 2 months for a $50 gift card to encourage participation.

#### 2.5.2 Qualitative Component

All PSWs/FPSWs, team leaders and managers from the 12 participating EIS, and the leaders of partner organisations are invited to participate in focus groups to ensure comprehensive representativeness. With the help of team leaders, purposive sampling was used to recruit youth, family members and clinicians, emphasising maximum variation across site‐level factors (i.e., rural/urban, academic/not, specific populations covered) and individual characteristics (i.e., age, gender and identification as ethnic, cultural, religious, or sexual minorities). Diversity in diagnoses and duration of follow‐up is also considered for youth and families. For clinicians, a variety of professional roles and training backgrounds (e.g., social workers, psychiatrists, nurses, occupational therapists) are considered to ensure an adequate representation of the clinical environment. Five to eight participants are targeted per stakeholder group, aligned with best practices for focus groups (Krueger and Casey 2014). The criterion of data saturation (Saunders et al. 2018) guides whether additional recruitment for focus groups is needed to ensure themes are fully explored and no new insights are emerging. Prior to enrolment, all focus group participants receive verbal and written information detailing the study objectives, the focus group procedures and how their contributions will inform our understanding of the implementation and impact of peer support.

### 2.6 Study Procedure and Data Collection

#### 2.6.1 Quantitative Component

Throughout the study period, data collection, analysis and feedback sharing with clinics occur in 4‐month cycles each from September 2023 (data collection of January 2024) until December 2026 (data collection of January 2027). Via the SARPEP data capturing platform (REDCap), EIS team leaders complete questionnaires on key program‐level practices regarding peer support (e.g., number of PSWs/FPSWs services offered/received, referral rates by clinicians), while PSWs/FPSWs and clinicians complete closed‐ended questionnaires on peer support integration and practices. Youth and family members can access SARPEP's ongoing surveys through QR codes, including a specific questionnaire on peer services allowing them to provide their self‐evaluation of the impact of peer support on their recovery and their satisfaction with services received at their convenience. Tools used for each stakeholder are detailed in Table 3.

**TABLE 3:** Mixed‐method matrix.

Research objective number 1: To evaluate the implementation of the PAIRPEP capacity building program as well as its impact on the integration process of peer support services in EIS, emphasising a comprehensive, multistakeholder perspective.		
**Quantitative research question**	**Stakeholder groups**	**Assessment Tools**
How do indicators related to the integration of peer support services evolve over time, and what specific trends can be identified in these changes? In what ways do varying contextual conditions impact the outcomes associated with peer support implementation?	PSWs	*Close‐ended and Likert scale co‐constructed questions to gather information on the following*: Practice of peer support within EIS: work schedule, modalities of interventions with youth, average number of meetings per patient, duration of follow‐up, types of support offer, source and type of referrals, tools used in relation to interventions, and inclusion of peer interventions in recovery plans
	FPSWs	
	Clinicians	*Likert scale co‐constructed questions to gather information on the following*: Integration of peer support interventions into the EIS service offer Role definition and role complementarity of PSW/FPSW Usefulness of PAIRPEP clinical tools
	Team Leaders	*Close‐ended and Likert scale co‐constructed questions to gather information on the following*: Overall rate of patients being offered and benefiting from peer support services Overall rate of clinicians recommending peer support services Access to information about peer support interventions and the role of PSWs Overall rate of utilisation of PAIRPEP clinical tools and formal inclusion of peer support interventions in recovery plans.
	Youth	*Likert scale questions co‐constructed to gather information on the following*: Complementarity of peer support services with other services Role clarity of the PSW/FPSW in the treatment plan
	Family members	*Likert scale questions co‐constructed to gather information on the following*: Complementarity of peer support services with other services Role clarity of the PSW/FPSW in the treatment plan
**Qualitative research question**	**Stakeholder groups**	**Assessment Tools**
How do stakeholders perceive and experience the implementation of peer support services at different stages of the process, and how do their perspectives evolve over time?	PSWs	*FG semi‐structured interview guide* Open‐ended questions assessing the experience of integrating peer support services into an EIS team, including challenges and factors that facilitate the integration journey, as well as feelings of inclusion and participation in decision‐making at both clinical and organisational levels.
	FPSWs	
	Clinicians	*FG semi‐structured interview guide* Open‐ended questions assessing the experience of integrating peer support services in the EIS service offer, including challenges and factors that facilitate the integration journey.
	Team Leaders	
	Managers	
	Users	*FG semi‐structured interview guide* Open‐ended questions on the experience of receiving peer support services and potential improvements to the peer support service offering in EIS.
	Family members	
	Partner organisations	

Research objective number 1A: To assess and refine a comprehensive capacity‐building program specifically designed to support the integration of peer services within EIS		
**Quantitative research question**	**Stakeholder groups**	**Assessment Tools**
What are the levels of stakeholder satisfaction with the tools and activities provided through the PAIRPEP project? To what extent are the tools and activities provided by the PAIRPEP project utilised by clinical teams within EIS settings?	PSWs	*Close‐ended and Likert scale co‐constructed questions to gather information on the following*: Training and feeling of competence: adequacy of the training and ongoing support, confidence in handling different aspects of clinical practice and various clinical situations Usefulness and utilisation of PAIRPEP clinical tools and activities.
	FPSWs	
	Clinicians	*Likert scale co‐constructed questions to gather information on the following*: Usefulness and utilisation of PAIRPEP clinical tools
	Team Leaders	*Likert scale and close‐ended co‐constructed questions to gather information on the following*: Rate of utilisation of PAIRPEP clinical tools.
**Qualitative research question**	**Stakeholder groups**	**Assessment Tools**
How do stakeholders perceive the tools and activities developed through the PAIRPEP project, and what specific suggestions do they have for their refinement?	PSWs	*FG semi‐structured interview guide* Open‐ended questions assessing specific activities, teaching and tools that have proved helpful in the integration of peer services in EIS, and soliciting feedback on PAIRPEP training, tools and activities, as well as aspects that need further refinement.
	FPSWs	
	Clinicians	
	Team Leaders	
	Managers	
	Partner organisations	
What factors influence the implementation of peer support services within the PAIRPEP initiative, and how do these insights inform potential refinements to the program?	Partner organisations	*FG semi‐structured interview guide* Open‐ended questions assessing factors influencing the integration of peer support services into EIS, including challenges in transitioning to this specific setting and factors facilitating this transition.

Research objective number 2: To evaluate the integration process of PSW/FPSW within EIS teams, as well as their impacts on the stakeholders involved.		
**Quantitative research question**	**Stakeholder groups**	**Assessment Tools**
What are the levels of satisfaction with peer services reported by users and family members, and to what extent do they believe that peer services support their recovery? How do indicators related to the integration of PSW/FPSW in the team evolve over time, and what specific trends can be identified in these changes?	Youth	INSPIRE‐Brief adapted tool to gather information on: Perceived impact of peer services on feelings of support, sense of hope, self‐esteem, engagement in meaningful activities, and sense of control over one's life Integration of the PSW/FPSW into the EIS team
	Family members	INSPIRE‐Brief adapted tool to gather information on: Perceived impact of peer services on feelings of support, sense of hope, self‐esteem, engagement in meaningful activities, and sense of control over one's life Integration of the PSW/FPSW into the EIS team
	Clinicians	*Likert scale co‐constructed questions to gather information on the following*: Perceived impact of peer support interventions on the recovery of the users and their families Integration of the PSW/FPSW into the EIS team
	PSWs	*Close‐ended and Likert scale co‐constructed questions to gather information on the following*: Integration within EIS: clarity of the role and recognition by the team, perception of integration (i.e., space to speak and ease of expressing opinions in meetings), consideration of the PSW's opinion, information sharing, clinical and emotional support.
	FPSWs	
**Qualitative research question**	**Stakeholder groups**	**Assessment Tool**
How do users and family members experience peer services, and in what ways they perceive these services influence their recovery process?	Users	*FG semi‐structured interview guide* Open‐ended questions on the experience of receiving peer services, including perceived benefits and aspects that did not meet expectations.
	Family members	
How do stakeholders perceive and experience the integration of PSWs/FPSWs into the EIS team at different stages of the process, and how do their perspectives evolve over time?	Clinicians	*FG semi‐structured interview guide* Open‐ended questions assessing the experience of integrating PSWs/FPSWs into the EIS team, including challenges and factors that facilitate the integration journey.
	Team Leaders	
	Managers	
What factors influence the integration of PSW/FPSW in EIS within the PAIRPEP project?	Partner organisations	*FG semi‐structured interview guide* Open‐ended questions assessing factors influencing the integration of PSWs/FPSWs into EIS, including challenges in transitioning to this specific setting and factors facilitating this transition.

Research objective number 2A: To assess the indirect impact of PSWs/FPSWs on EIS team		
**Quantitative research question**	**Stakeholder groups**	**Assessment Tools**
To what extent do peer services impact the workload?	Clinicians	*Likert scale co‐constructed questions to gather information on the following*: Perceived impact of PSW on workload
**Qualitative research question**	**Stakeholder groups**	**Assessment Tools**
How does the implementation of peer services influence and shape the work dynamics and service offer within EIS?	PSWs	*FG semi‐structured interview guide* Open‐ended questions exploring how a project like PAIRPEP and the presence of PSWs/FPSWs might impact team dynamics, service offerings, and the overall delivery of services within EIS.
	FPSWs	
	Clinicians	
	Team Leaders	
	Users	
	Family members	

Abbreviations: EIS: early intervention services, FG: focus groups, FPSWs: family peer support workers, PSWs: peer support workers.

#### 2.6.2 Qualitative Component

Focus groups are planned to be conducted online (Zoom Health) around the project's onset; after about 12 months; and between 24 months and the project ending, with all stakeholder groups to capture the evolution of the implementation process. Each session (90 min) is conducted in French and co‐facilitated by trained moderators. For focus groups involving PSWs and FPSWs, a researcher with expertise in focus groups co‐facilitates with a person with lived experience and peer support experience, ensuring sensitivity to positionality issues within the group. For focus groups involving EIS clinicians, managers, users, family members and community organisation representatives, two members of the research team with experience in group facilitation and dynamics co‐facilitate each session. When possible, at least one facilitator from a minority background is prioritised to promote inclusive participation.

### 2.7 Assessment Tools

The selection and development of assessment tools was preceded by a preparatory phase aimed at gathering insights into frameworks for evaluating the integration of peer support in EIS and youth‐oriented services. This groundwork involved a review of the existing literature as well as consultations with research groups and researchers/research faculty with lived experience in Canada, the USA and Australia that have been integrating peer support into EIS or integrated youth services, and six Quebec community organisations specialised in peer support. Building on this foundation, the assessment tools were developed through a multi‐phase, iterative co‐design process that actively engaged clinicians with diverse expertise relevant to EIS and peer support, academic researchers and individuals with lived experience (patients and family members, both with and without prior EIS involvement). Based on discussions with all stakeholders, initial drafts of quantitative questionnaires and qualitative interview guides were prepared by researchers with relevant expertise and subsequently reviewed and refined during monthly multidisciplinary meetings as well as a few full days of meetings of the core research team (which includes 3 PSW and 1 FPSW) until internal consensus was reached. Each draft instrument was then pilot‐tested with one or more advisors from each stakeholder group (who were not subsequently enrolled as study participants), and their feedback was systematically incorporated to improve clarity, relevance and acceptability. The final assessment tools aligned with the research objectives are presented in the mixed‐methods matrix in Table 3.

#### 2.7.1 Quantitative Component

The questionnaires consist of closed‐ended items with Likert scales, tailored to each group. To assess the impact of peer services on recovery for the youth and family members, the INSPIRE‐Brief questionnaire (Moeller et al. 2024) was selected following a literature review and consultation with peer support experts and researchers.

#### 2.7.2 Qualitative Component

The focus group interview guides follow a semi‐structured format with open‐ended questions, fostering rich, open dialogue and allowing for the emergence of unanticipated insights. The group interview guides for each stakeholder group are grounded in the core elements of the MRC framework. While core themes are consistent across groups to enable comparisons, each guide is adapted to reflect the specific perspectives of the various stakeholders. Key topics explored include: (i) the context of each EIS team and how the integration of peer support takes shape within the team, including suggestions to create the most conducive environment for peer service implementation; (ii) feedback on PAIRPEP training, tools and activities, with recommendations for refinement to better meet the needs of both the EIS teams and the PSWs; (iii) stakeholder contributions, roles and degree of engagement in implementation processes; (iv) resources required for effective implementation and long‐term sustainability of peer support and (v) the perceived impact of peer support on youth, families, clinical teams and the broader EIS community. The mirrored structure of the interview guides is intentionally designed to enable comparison of experiences across groups, capturing both shared and divergent perspectives among stakeholders (e.g., how the experience of PSWs integrating into a new team is similar to or different from that of FPSWs), as well as the ways in which these experiences evolve over time.

### 2.8 Data Analysis

#### 2.8.1 Quantitative Component

Using R and SPSS software, we will examine the relationships between contextual factors (e.g., academic vs. non‐academic, urban vs. rural) and key outcome variables, such as satisfaction with services or peer service utilisation rates. Appropriate descriptive and inferential statistics will be conducted (e.g., Chi‐square or Fisher's exact tests for categorical variables, *T*‐tests or Mann–Whitney *U* tests for continuous variables). To assess trends over time, mixed‐effects models will be applied, accounting for the repeated measures design. These models will facilitate the evaluation of changes in key outcome variables across 4‐month intervals, while adjusting for contextual factors.

#### 2.8.2 Qualitative Component

Audio recordings are transcribed and thoroughly reviewed to extract and identify key themes with NVivo software. Thematic analysis follows a structured approach, including familiarisation with transcripts, coding, theme identification, theme review, definition and report writing. Themes are refined through iterative discussion among co‐researchers to reach consensus (Braun and Clarke 2006). Thematic analysis is initially conducted separately for each stakeholder group to identify similarities, differences and complementarities across perspectives. Each time point is treated initially as an independent study (Saldana 2003). Recurrent cross‐sectional thematic analysis is then utilised rather than longitudinal analysis to compare findings from different time points (Grossoehme and Lipstein 2016). This approach accommodates potential variability in participant composition over time (Grossoehme and Lipstein 2016). The results are refined through ongoing discussions among researchers to reach consensus.

### 2.9 Data Triangulation

Triangulation is achieved by integrating qualitative and quantitative data from diverse stakeholder perspectives (Fielding et al., Fielding 2012). Qualitative and quantitative findings are systematically compared using joint display tables to identify convergences (which strengthen validity) and divergences (which are explored through discussions with the research team and stakeholders). The inclusion of dual data sources and diverse stakeholder perspectives enhances both the depth and comprehensiveness of the findings, providing a nuanced understanding of peer support integration across the EIS.

### 2.10 Ethics and Dissemination

The study is conducted in accordance with the principles of the Declaration of Helsinki. Approval was obtained from the Centre de Recherche du Centre hospitalier de l'Université de Montréal (CRCHUM) Ethics Committee (project number MP‐02‐2020‐8627). Informed consent is obtained from all focus group participants prior to study initiation, and participants may withdraw their consent at any time. The ethics committee has authorised the use of quantitative data (completed anonymously and analysed as aggregated data) without a signed consent form, as people de facto consent to the RLHS quality improvement process when participating in the SARPEP online questionnaire (an explanation is provided on the electronic data capture platform before completing the survey).

Results are disseminated to the SARPEP community through data visualisation feedback reports embedded in the SARPEP RLHS and the community of practice exchange sessions, including all stakeholders, notably decision makers. Data will be shared with the scientific community through publications and conference presentations.

## 3 Results and Conclusions

PAIRPEP will contribute to the growing body of knowledge on peer support integration in EIS and mental health services and on the advantages of co‐designing research and interventions with persons with lived experience. A distinctive feature of the project is its extensive co‐design process, which systematically engaged stakeholders throughout the development of both the PAIRPEP intervention program and its mixed‐methods evaluation protocol. This approach, which fosters shared ownership and collaborative learning from the outset, not only ensures relevance and feasibility but also integrates knowledge translation and application into practice and policy, facilitating sustainability. Its participatory design, embedded in a real‐world clinical framework and RLHS, aligns with international best practices and offers valuable insights for stakeholders involved in youth mental health care. Through the successful integration of new lived experience team members and the integration of a new intervention fostering hope and promoting a recovery‐oriented culture within EIS, PAIRPEP is expected to positively impact EIS and their stakeholders.

PAIRPEP is expected to improve peer support accessibility, foster youth and family engagement and their recovery. Clinicians should benefit from structured guidance and tools to facilitate inclusion of peer support services in their practice throughout the patient's care trajectory. For PSWs and FPSWs, PAIRPEP will provide specialised training, supervision and mentorship to ensure role clarity, easier integration within teams, professional development and sustainability.

At the organisational and systemic levels, local EIS and the MSSS will benefit from the data‐driven refinement processes and community‐of‐practice exchanges. Policymakers may draw on study findings to inform the development of policies supporting sustainable peer support infrastructures and funding mechanisms. PAIRPEP could serve as a model for other mental health programs aiming to integrate peer support services.

Overall, PAIRPEP aims to establish a sustainable and adaptable model for peer support integration in EIS, addressing critical implementation gaps. This implementation study will enhance understanding of the structural, relational and contextual factors shaping integration processes. Its mixed‐method, longitudinal and feedback‐informed design may serve as a template for future evaluations of newly developed complex mental health interventions, supporting practices, training and policy development grounded in recovery‐oriented values. Finally, using our RLHS approach for evaluating a complex multifaceted capacity‐building program may be an exemplar of the role a RLHS can play in supporting the implementation of best practices through continuous adaptation and improvement fostered by real‐time data sharing and stakeholder engagement.

## Ethics Statement

This study has received ethics approval from the Research Ethics Committee of the Centre de recherche du CHUM (reference: MP‐02‐2020‐8627).

## Conflicts of Interest

The authors declare no conflicts of interest.

## Data Availability

As a protocol article, no data are currently available. Upon study completion, the possibility of data sharing will be assessed based on ethical approval, institutional policies and the specific consent conditions provided by participating Early Intervention Services and stakeholders. No identifiable or individual‐level data from participants will be shared without explicit consent.
